# Influenza virus Matrix Protein M1 preserves its conformation with pH, changing multimerization state at the priming stage due to electrostatics

**DOI:** 10.1038/s41598-017-16986-y

**Published:** 2017-12-01

**Authors:** Eleonora V. Shtykova, Liubov A. Dadinova, Natalia V. Fedorova, Andrey E. Golanikov, Elena N. Bogacheva, Alexander L. Ksenofontov, Liudmila A. Baratova, Liudmila A. Shilova, Vsevolod Yu. Tashkin, Timur R. Galimzyanov, Cy M. Jeffries, Dmitri I. Svergun, Oleg V. Batishchev

**Affiliations:** 1 Shubnikov Institute of Crystallography of Federal Scientific Research Centre “Crystallography and Photonics” of Russian Academy of Sciences, Moscow, Russia; 2 Semenov Institute of Chemical Physics, Russian Academy of Sciences, Moscow, Russia; 3 Belozersky Institute of Physico-Chemical Biology, Moscow State University, Moscow, Russia; 4 Frumkin Institute of Physical Chemistry and Electrochemistry, Russian Academy of Sciences, Moscow, Russia; 5 Moscow Institute of Physics and Technology, Dolgoprudniy, Russia; 6 National University of Science and Technology “MISiS”, Moscow, Russia; 7 European Molecular Biology Laboratory, Hamburg Outstation, c/o DESY, Hamburg, Germany

## Abstract

Influenza A virus matrix protein M1 plays an essential role in the virus lifecycle, but its functional and structural properties are not entirely defined. Here we employed small-angle X-ray scattering, atomic force microscopy and zeta-potential measurements to characterize the overall structure and association behavior of the full-length M1 at different pH conditions. We demonstrate that the protein consists of a globular N-terminal domain and a flexible C-terminal extension. The globular N-terminal domain of M1 monomers appears preserved in the range of pH from 4.0 to 6.8, while the C-terminal domain remains flexible and the tendency to form multimers changes dramatically. We found that the protein multimerization process is reversible, whereby the binding between M1 molecules starts to break around pH 6. A predicted electrostatic model of M1 self-assembly at different pH revealed a good agreement with zeta-potential measurements, allowing one to assess the role of M1 domains in M1-M1 and M1-lipid interactions. Together with the protein sequence analysis, these results provide insights into the mechanism of M1 scaffold formation and the major role of the flexible and disordered C-terminal domain in this process.

## Introduction

Influenza viruses belongs to the most widespread and potentially dangerous pathogenic group of viruses in the world^1^. Influenza A is an enveloped virus belonging the *Orthomixoviridae* family^2^. Its outer envelope is formed by host cell derived bilayer lipid membrane, BLM, containing incorporated glycoproteins haemagglutinin (HA) and neuraminidase (NA) along with the proton channel M2. The inner shell of the virion is represented by a membrane-associated scaffold of matrix proteins, M1, that makes contacts with both viral ribonucleoproteins, RNP, and lipid envelope with the cytoplasmic tails of HA and NA^3^. Influenza virus penetrates the cell using predominantly endocytosic pathway^4^, resulting in the virion entering into the endosome. A pH drop inside the late endosome triggers the fusion of the viral and endosomal membranes^5^. Under these conditions, the first step is a priming stage^6^ at a pH of around 6.0 that causes a possible conformational change in M1 protein and the subsequent dissociation from the viral RNP^6–8^ resulting in a loss of viral particle rigidity^9^. During this process, M1-lipid interactions remain intact^10^. A conformational change in HA at pH 5.5 leads to a tight contact of the viral and endosomal membranes and formation of the small fusion pore^11,12^. Freezing of the system at the given pH value reveals no change in the viral envelope and only the target membrane is deformed under action of viral fusion proteins^12^. Further decreases in pH results in the acidification of the medium inside the viral particle due to the action of proton channels M2. This process leads to a disintegration of the viral protein scaffold resulting from the increased charge of M1 monomers in acidic medium^10^, and their partial dissociation from the viral lipid envelope^13^. Finally, these processes widen the fusion pore facilitating the entry of the viral RNP into the cytoplasm of the infected cell^3^. We have recently demonstrated that M1 protein may also contribute to this stage by inducing a lateral tension in the viral membrane^10^. Therefore, the M1 matrix protein, apart from being a mechanical skeleton of the virion, acts as a crucial factor for different viral processes during infection.

Isolated M1 protein in solution at low pH demonstrates a level of structural anisotropy that is characterized by a globular core (NM-domain, amino acids 1–164) and an extended flexible tail (C-terminal domain, amino acids 165–252)^14,15^. The X-ray crystallography atomic structures of the isolated NM-domain^16–19^, show that the overall globular domains form dimers in the crystal lattice at both acidic and neutral pH, but with different orientations of monomers in the asymmetric crystal unit^17,19^. Solution studies of the full-length protein using dynamic light scattering and small-angle neutron scattering indicated that M1 adopts an elongated monomeric form^18^ at neutral pH, while our previous small angle X-ray scattering experiments^14^ showed full-length M1 at pH 4.7 is predominantly monomeric with small fraction of the protein in the form of helical oligomers, close to those observed in intact viral particles at neutral pH^20,21^. Of note, the models obtained from the SAXS experiments of monomeric M1, that are typified by a globular domain with a flexible extended tail, are consistent with the high-resolution X-ray crystal structure of full-length M1 from salmon anemia virus (PDB: 5WCO^22^). However, what the high-resolution model does not capture is the propensity of M1 to self-associate into clusters^14^ and respond to changes in pH, for example, the pH-dependent oligomerization of NM-domain and the dimerization of the C-terminal domain have been previously reported^23^. Thus, the change in protein-protein interactions with pH, as well as possible conformational changes in the individual M1 molecule remains enigmatic especially in context of additional interactions of the protein with itself and other viral constituents, for example lipids.

In a number of publications^24–27^, M1-lipid interactions are suggested to be predominantly electrostatic in nature, with the phosphatidylserine as the main partner in lipid membrane. The same forces are expected to be pivotal for conformational changes of M1 with pH^19^. To perform its functional role, M1 contacts both the viral RNP and the lipid envelope^17^. As demonstrated in^6^, the matrix protein interacts with the viral RNP most likely via the C-terminal domain, while the binding of M1 to the lipid bilayer occurs via the NM domain. In^16^ authors analyze the X-ray crystal structures of N-terminal domain of M1 protein and hypothesize the insertion of this domain into the viral lipid membrane. This insertion may be possible, as it was shown later, due to the presence of amphipathic helices in the N-terminal domain structure^28^. The possibility hydrophobic forces being involved in the interactions of M1 with lipid membranes is also mentioned in^29^ based on the emission spectrum of the fluorescent probe 12-(9-anthroyl)-stearic acid incorporated into viral particles with removed glycoprotein spikes. The same conclusions are obtained from the solubilization of M1 protein with liposomes^30,31^ and the study of the adsorption of M1 on uncharged lipid monolayers^32^. These results suggest that although electrostatic forces are a main contributor to M1-M1 and M1-lipid interactions and their modulation with pH, some charge-independent forces are also responsible for the interaction of the protein with itself and with lipid membranes.

In the present work, we combined two structural methods, small angle X-ray scattering (SAXS) and atomic force microscopy (AFM), with complementary techniques, to clarify the nature of M1-M1 interactions and their change with pH. We suggest a model of pH-dependent oligomerization of M1 and calculate the energy of the interaction of the individual M1 molecules as well as an estimate of M1 protein charge at different values of pH.

## Materials and Methods

### Influenza A virus preparation

Influenza virus strain A/Puerto Rico/8/34 (subtype H1N1) was propagated in 10-day-old embryonic chicken eggs and purified by centrifugation through 20% (vol/vol) sucrose in STE buffer (100 mM NaCl, 10 mM Tris-HCl, and 1 mM EDTA, pH 7.4) at 21,000 rpm for 90 min at 8 °C in the SW 27.1 rotor of a Beckman-Spinco L5-75 centrifuge, as described in^33^.

### Isolation of the M1 protein

The protein was isolated from intact influenza A/Puerto Rico/8/34 virions as described previously in^34^. The purity of the protein samples was determined by size exclusion chromatography, Coomassie and silver stained SDS-PAGE^35^ and trypsin in-gel hydrolysis/MALDI-TOF mass spectrometry. For further investigations, the M1 protein solution was dialyzed by Bio-Beards in 100 mM NaCl/20 mM MES buffer at pH 4.0.

### M1 preparation for measurements at different pH

A general protocol for the preparation of full-length M1 samples for measurements at different pH consisted of the following steps that were performed, wherever possible, at 10 °C:

1. The pH of the solution containing M1 from pH 4.0 up to 7.85 and back was performed by alkali (NaOH) and acid (HCl) titration, respectively.
2. Each pH adjusted sample was incubated for one hour at room temperature to reach necessary pH condition.
3. To remove possible insoluble M1 sediments before measurements the samples were centrifuged using Beckman-Coulter Allegra X-22R self-cooling high-speed centrifuge at 10 °C, 14000 rpm for 10 minutes. Taking into account that the approximate quantity of M1 precipitate did not depend on pH, we assume that the baseline amount of sediment was caused by the natural sedimentation of the protein during short manipulations with it at room temperature.
4. The protein concentration of each pH-adjusted sample was measured using a Thermo Scientific NanoDrop ND-1000 Spectrophotometer with an Abs 280 nm extinction coefficient, E_0.1%_, of 0.476 for 1 mg/ml solution (calculated using ProtParam^36^).

### Scattering experiments and data analysis

Synchrotron SAXS measurements were performed at the European Molecular Biology Laboratory (EMBL) on the storage ring PETRA III (DESY, Hamburg) at the EMBL-P12 beam line equipped with a robotic sample changer and a 2D photon counting pixel X-ray detector PILATUS-2M detector (DECTRIS, Switzerland). The scattering intensity, *I*(*s*), was recorded in the range of momentum transfer 0.08 < *s* < 4.5 nm^−1^, where *s* = (4*πsinθ*)*/λ*, 2*θ* is the scattering angle, and *λ* = 0.124 nm is the X-ray wavelength^37^. The measurements were carried out in 100 mM NaCl, 20 mM MES buffer in pH range from 4.0 up to 6.8–7.0, at 10 °C using continuous flow operation over a total exposure time of 1 s collected as 20 × 50 ms individual frames to monitor for potential radiation damage (no radiation effects were detected)^38^. The data were corrected for the solvent scattering and processed using standard procedures^39,40^ with additional data analysis performed using the program PRIMUS^41^. To account for interparticle interactions, we measured and compared samples of M1 between 1.5–4.0 mg/ml. No concentration dependence was observed, thus, for the data interpretation and modeling the scattering curves with maximal concentrations were used to reduce influence of the experimental noise.

The values of the forward scattering and radii of gyration *R*_*g*_ were calculated from the experimental SAXS patterns using Guinier approximation,

1 $${I}_{\exp }(s)=I(0){\exp }(-{s}^{2}{R}_{g}^{2}/3),$$

which is valid in the range of (*sR*_*g*_) approximately <1.3^42^. These parameters and the maximal diameter of the particle *D*_*max*_ were also computed from the distance distribution function *p(r)*. The latter was evaluated by the program GNOM^43^ using Eq (2)

2 $$p(r)=\frac{1}{2{\pi }^{2}}{\int }_{0}^{\infty }srI(s){\sin }(sr)ds$$

The low-resolution shapes were reconstructed by the *ab initio* method, DAMMIN^44^, employing a dummy atom (bead) model of a particle. Starting from a random assembly, both programs utilize simulated annealing (SA) to build models fitting the experimental data *I*_exp_(*s*) with minimal discrepancy

3 $${\chi }^{2}=\frac{1}{N-1}\sum _{j}{[\frac{{I}_{{\exp }}(s{}_{j})-c{I}_{calc}({s}_{j})}{\sigma ({s}_{j})}]}^{2},$$

where *N* is the number of experimental points, *c* is the scaling factor and *I*_calc_(*s*_*j*_) and *σ*(*s*_*j*_) are the calculated intensity from the model and the experimental error at the momentum transfer *s*_*j*_, respectively.

An alternative hybrid approach was applied to reconstruct approximate conformations of the missing C-terminal domain of M1 accounting for the high-resolution structure of the NM domain (PDB entry 1AA7^16^). The program CORAL^39^ was used to optimize the spatial position of the C-terminal domain, represented as a chain of dummy residues connected to the NM-doamin against the full-length protein SAXS data at different pH. For both *ab initio* and hybrid modeling, multiple reconstructions were performed, which yielded consistent models. The outputs were analyzed using programs SUPCOMB^45^ and DAMAVER^46^ to identify the most typical models of the protein or its clusters in solution.

The flexibility of the C-terminal domain, which secondary structure elements was predicted earlier^15^, has been quantitatively analyzed by the ensemble optimization method (EOM)^47^. This method selects an ensemble of possible conformations from a pool of randomly generated models, in this instance using the crystal structure of the NM domain with a randomly generated C-terminal region of the M1 protein. CRYSOL^48^ was used to calculate the theoretical scattering from these models and a genetic algorithm (GAJOE) was employed to select ensembles of conformations whose combined-weighted scattering best fit the experimental data.

To analyze the amount of aggregates in the M1 solutions we used the program OLIGOMER^49^. Given the scattering intensities of components in a mixture, *I*_*i*_(*s*), the program fits the experimental scattering curve by their linear combination to determine their fractions *w*_*i*_. The equation

4 $$I(s)=\sum ({w}_{i}\times {I}_{i}(s))$$

is solved with respect to *w*_*i*_ by non-negative least-squares to minimize the discrepancy between the experimental and calculated scattering curves.

### Docking procedure

In order to obtain the full-length structures of the M1 protein docking simulation was performed using ZDOCK protein-protein docking server for complex prediction^50^. High-resolution structures of NM-domain monomers obtained from the corresponding dimeric X-ray crystal structures (PDB entries: 1AA7^16^ and 1EA3^18^), were docked against C-terminal domain whose high-resolution structure was predicted by ROSETTA modeling software^51^ (and is consistent with PDB: 5WCO^22^). The algorithm of the ZDOCK 3.0.2 software is based on the rigid-body protein docking using Fast Fourier Transform approach. This program has a scoring function that includes shape complementarity, electrostatics, and a pairwise atomic statistical potential^52^. The ZDOCK output represents the top 10 models of the predicted complexes^50^ and the selection of the best models produced was performed by fitting calculated scattering from these models to the experimental SAXS data. The structure of the M1 monomer with the predicted C-domain structure was submitted as a receptor and a ligand, respectively. Structural analysis was conducted by selecting the binding site residues (the amino acid 158 for monomeric structures and the amino acid 1 for C-terminal domain) when applying other ZDOCK default parameters.

### Atomic force microscopy (AFM)

Structures formed by M1 protein upon adsorption on a negatively charge mica surface was studied on the Multimode Nanoscope IV setup (Veeco Digital Instruments, USA) equipped with E type scanner and electrochemical fluid cell. All experiments were carried out in tapping mode at room temperature in working buffer solution. For scanning, SiN_3_ cantilevers were used with nominal spring constant of 0.06 N/m (type SNL, Bruker, USA) with a tip radius of approximately 2 nm. A protein concentration of 2 × 10^−3^ mg/ml was used in 100 mM NaCl, 50 mM MES buffer, which corresponds to the dense protein monolayer at pH 7.1^10^. In some experiments the concentration of NaCl was 50, 150, 200 or 250 mM. The value of pH was varied from 4.0 to 7.1 with a step of 0.5 pH increments. A 200 µl droplet of protein solution with given pH value (or NaCl concentration) was applied on the surface of the disk of freshly cleaved mica (Veeco Digital Instruments, USA). After half an hour of adsorption at room temperature, the sample was placed into an AFM cell filled with working buffer solution and scanning commenced. Image processing as well as estimation of surface coverage were made with WSxM software^53^.

### Zeta Potential Measurements

Zeta potential measurements were carried out using a ZetaSizer Nano ZS instrument (Malvern Instruments Ltd., UK) with the corresponding proprietary software. All measurements were performed at 18 °C with seven measurements, each comprising 20 runs. The M1 concentration used for the experiments was 0.05 mg/ml. The measurements were carried out in 5 mM NaCl, 2.5 mM MES buffer in range of pH from pH 4.6 up to 7.3.

### Intramembrane Field Compensation (IFC)

IFC measurements of M1 adsorption and desorption were performed as described in^10^. In brief, free-standing bilayer lipid membranes (BLM) were formed by Mueller-Rudin technique^54^ at the small aperture (diameter is 0.8 mm) in a septum dividing two 500 μl chambers of the Teflon cell. The chambers were filled with working buffer solution of 20 mM KCl, 0.1 mM EDTA, 5 mM sodium citrate, and pH 4.0, 5.0, 6.0 or 7.0. BLM were made from a solution of 30 mol. % of diphytanoylphosphatidylserine, DPhPS, and 70 mol. % of diphytanoylphosphatidylcholine, DPhPC (Avanti Polar Lipids, USA) in decane (Sigma, USA) with total lipid concentration of 15 mg/ml. The adsorption of M1 protein on the one side of BLM changed the difference of boundary potentials across the lipid bilayer, which was measured using IFC technique^55^. The washing of M1 protein from the cell was performed by perfusion with protein-free buffer solution using peristaltic pump (LKB, Sweden). Protein was added to the BLM in concentration of 2 × 10^−3^ mg/ml at given pH value, and after reaching the stationary level of the difference of boundary potentials, perfusion was started. The difference between the stationary levels after and before the start of perfusion divided by the latter level and multiplied by 100% was treated as a percent of desorbed protein. Although the theory of the IFC method can explain in detail only the adsorption of small molecules, reliable results can be obtained for large protein molecules as well^56–58^, assuming that the measured potential difference, Δ*φ*, is directly proportional to the amount of the protein adsorbed per unit area of the membrane. Recently we have shown that the same approach is also justified for M1 protein^10^.

## Results and Discussion

### Primary analysis of the scattering curves

M1 protein forms a viral scaffold at neutral pH of the cell cytoplasm and isolated M1 can be soluble without detergents only in acidic medium^34^. Therefore, it is reasonable to expect that the number of M1 aggregates will increase with pH. To remove possible M1 sediments the samples at different pH, the samples were centrifuged before SAXS measurements and then concentration of the solutes was checked. We expected more sediment at higher pH, but it was found that the amount of the sediments practically did not depend on pH (data not shown). The concentration of the protein in solution changed slightly after centrifugation, but equally in all test-tubes. This observation suggests that increasing the pH does not lead to the severe non-specific and insoluble aggregation of M1.

**Figure 1:**
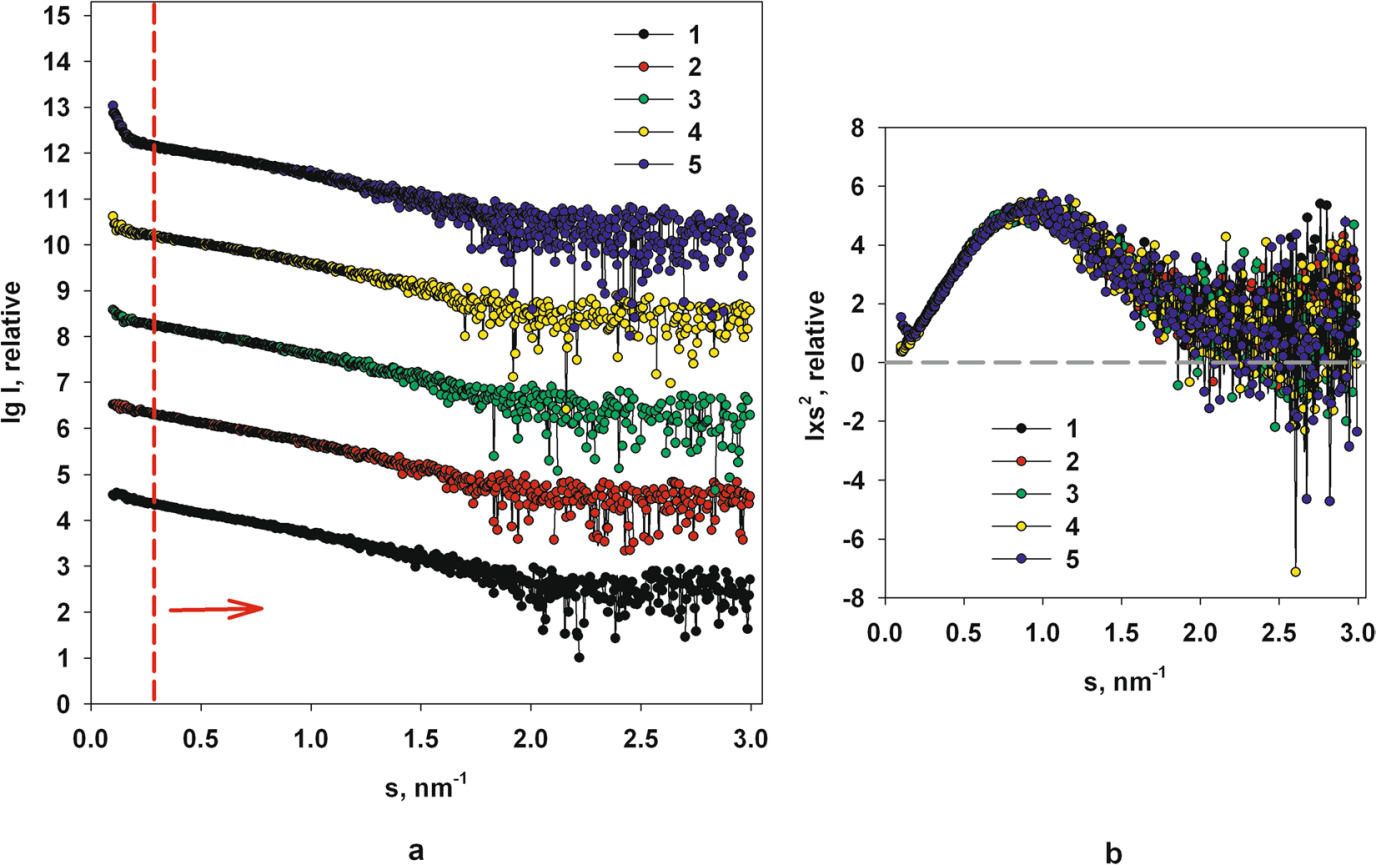
Experimental SAXS curves from the full-length M1 protein with gradually increasing pH: (**a)** 1 − pH 4.0; 2 − pH 5.2; 3 − pH 6.0; 4 − pH 6.4; 5 − pH 6.8. The curves are shifted along the ordinate by one logarithmic unit for better visualization. The red dashed line separates regions of scattering from the M1 self-assembles (to the left from the line), and from the individual M1 macromolecules (to the right from the line). (**b)** The Kratky plots of the all specimens (the data was normalized against the solute concentration). All symbols and numbering are the same as on the panel a.

Figure 1a shows the experimental SAXS data measured from the M1 protein upon increasing the pH in solution from 4.0–6.8. As is observed in Fig. 1a, the scattering curves at all pH values reveal an upturn at very small angles in the range of the scattering vectors *s* < 0.3 nm^−1^. This fact points to the formation of soluble M1 protein assemblies co-existing in solution with individual M1 monomers even at acidic pH, as it was observed previously^14^. The scattering curves also reveal the growth of the amount of associates with pH given that the upturn of the scattering intensity at very small angles becomes higher with increasing pH.

To understand the nature of the pH-based phenomenon of assembly formation we performed separate analysis of the structure of isolated M1 and its associates at systematically increasing values of pH applying the same protocol as in^14^. First, we modelled the structure of individual M1 monomers from the truncated SAXS curves in the scattering vector range 0.3 < *s* < 3.5 nm^−1^. Then, to obtain the scattering from the soluble M1 clusters, the scattering from the individual M1 particles (computed from the first stage of the structural analysis, see below) was subtracted from the experimental data of the full-length protein. The resulting difference curve was then used to model the low-resolution structure of the M1 clusters in solution. This method of analysis is justified as large clusters contribute mostly to the scattering intensities at very small angles^58^. Indeed, and in the specific case of M1, the similarity between of all the scattering data for scattering vectors *s* > 0.3 nm^−1^ suggests that the influence of M1 assemblies is negligible at higher scattering angles and therefore data above *s* > 0.3 nm^−1^ can be employed to analyze the structural properties of M1 monomers. For example, the scattering data in the interval 0.3 < *s* < 3.5 nm^−1^ demonstrate a good agreement in the respective Kratky plots (Fig. 1b) suggesting that the overall structure of the M1 monomer is well preserved across the pH values. The Kratky plots of all specimens (Fig. 1b) have a characteristic bell-shaped appearance indicating that all samples are mostly folded^39,59–63^.

**Figure 2:**
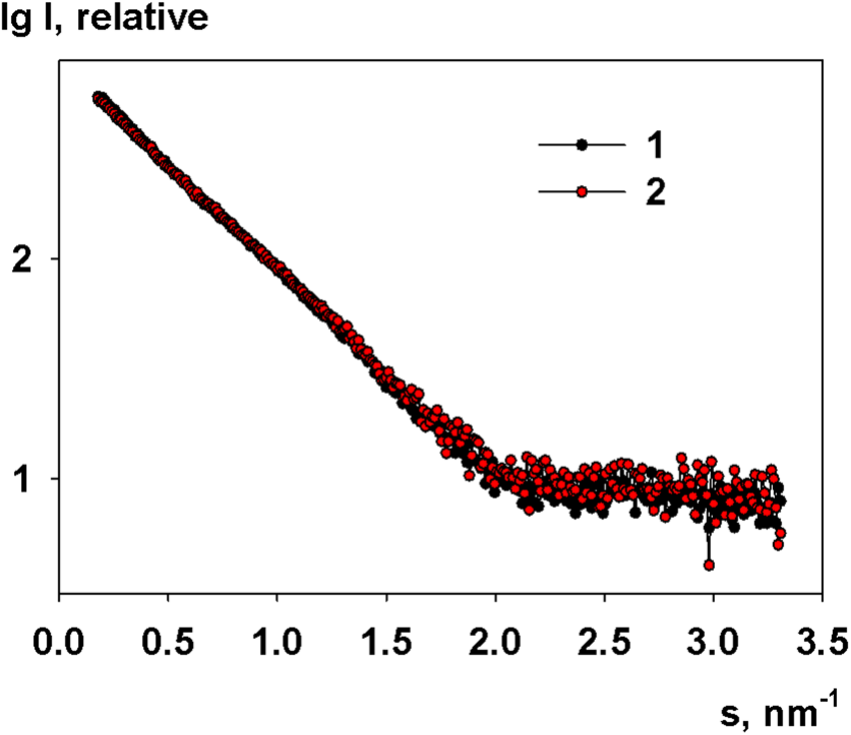
Experimental scattering curves at pH 4.0: 1 - initial SAXS curve at pH 4.0, 2 - SAXS curve after titration from pH 6.8 to pH 4.0.

Gradually decreasing the pH of the solutions back to acidic conditions did not result in any major changes of the scattering data except for the level of assemblies in solution. The sharp upturn of the scattering intensities at very small angles consistently became lower during the stepwise acidification of the M1 samples. Figure 2 demonstrates a good agreement between the scattering curves from the M1 protein at pH 4.0 before and after pH cycling (up to neutral pH conditions and back to the acidic pH). To infect the cell the virus protein scaffold should be destroyed^64^. It happens when the interior of the virus is acidified by the action of M2 channels. Our study demonstrates that the structure of M1 monomers does not change after the acidification and the virion decomposition. Moreover, the agreement between the scattering curves at the initial pH and after the re-acidification indicates that the association process is reversible.

### Shape restoration of the full-length M1 protein and modelling of C-terminus at different pH

**Figure 3:**
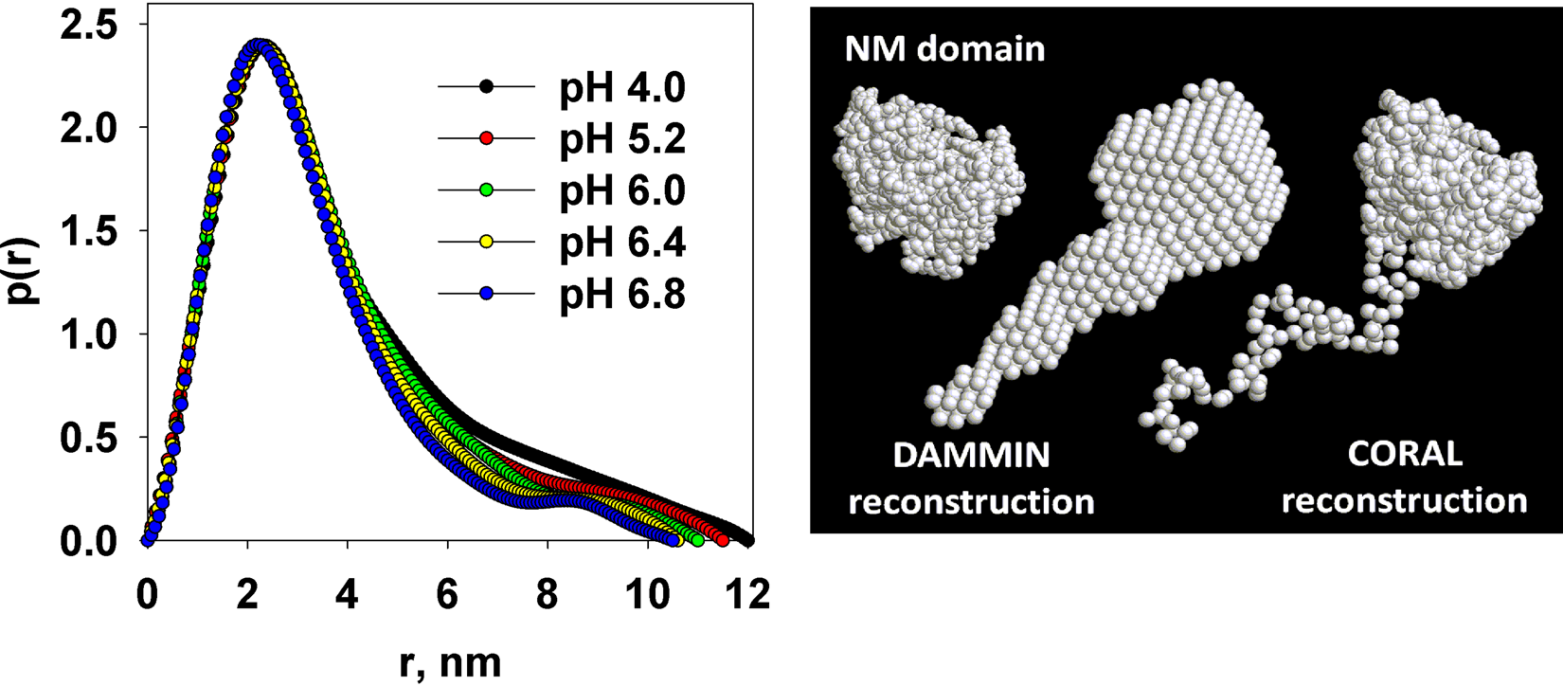
Shape reconstruction of M1: distance distribution functions *p*(*r*) at different pH (left panel); the *ab initio* DAMMIN reconstruction of the full-length M1 and CORAL hybrid modeling of the full-length M1 consisting of the high-resolution X-ray crystal structure of the NM-domain connected to C-terminal domain represented as a string of dummy amino acids (right panel).

In order to prove the preservation of the full-length M1 protein structure with changes in pH, we employed *ab initio* shape reconstruction and CORAL hybrid modelling. The probable real-space distance distribution functions, *p*(*r*), of M1 at different pHs were computed by GNOM using the intensities truncated to *s*_*min*_ = 0.3 nm^−1^. The calculated *p*(*r*) functions yield the maximum size *D*_*max*_ in the range from 10.5 to 12 nm and the *R*_*g*_ between 2.9 and 3.1 nm, agreeing well with the earlier results^14^. Figure 3 displays the *p*(*r*) profiles normalized to the same maximum value, which essentially coincide up to r ≈ 4 nm indicating that the core part of M1 remains unchanged. At larger distances, the *p*(*r*)’s for higher pH show somewhat lower values possibly indicating that the C-terminal tends to be more compact with increasing pH.

Using Guinier approximation with *sR*_*g*_ limits from 0.85 to 1.30^42^ we obtained the same value of *R*_*g*_ averaged over all experimental SAXS patterns for gradually changed pH of M1 solutions. The excluded (Porod) volumes of the isolated M1 macromolecules ranged from 52 to 63 nm^3^ at different pH. The averaged value of this parameter was found to be 54 ± 6 nm^3^. Since the empirical ratio between Porod volume and molecular mass of a protein is about 1/(~1.7)^63^, the estimated molecular mass of M1 corresponds to the monomeric form of the protein at any pH values studied in the present work.

Both *ab initio* and hybrid modelling approaches at different pH revealed the same structural anisotropy of the protein as has been observed previously^14^. The M1 macromolecule possesses a globular NM-domain and an extended and potentially flexible C-terminal domain. Importantly, the restored shapes were very similar to each other at all pH values (Fig. 3, right panel). Thus, we conclude that pH of the environment does not grossly affect the conformation of the compact NM domain while the C-terminal part stays flexible in solution.

**Figure 4:**
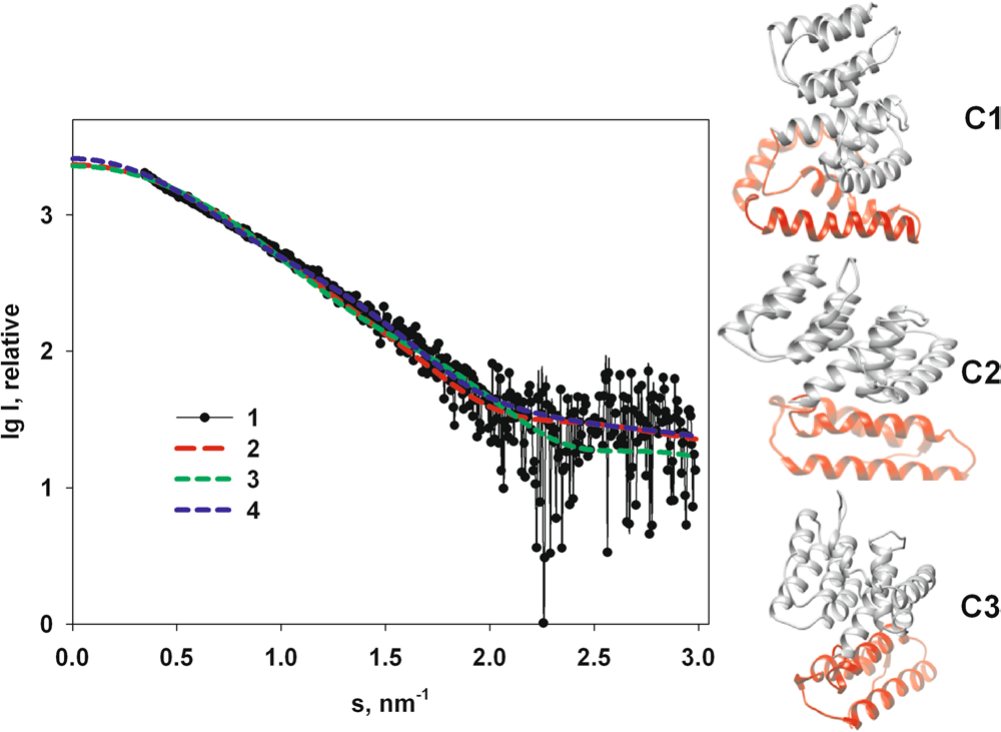
Experimental data from M1 (1) and calculated scattering curves from models of the most relevant high-resolution models of full-length M1 predicted by ROSETTA: C1 - curve 2, C2 - curve 3 and C3 - curve 4.

Both the dummy atom representation and hybrid model of the protein only provide low resolution structures of the full-length M1 protein in solution. We further attempted to construct a quazi-atomic model of the full length M1. For this purpose, the tentative high-resolution structures of the C-terminus were predicted by ROSETTA^49^, and docked against the NM-domain using ZDOCK protein-protein docking server for complex prediction^50^. To narrow down the diverse structures predicted by ROSETTA, tritium bombardment data^15^ were used to obtain the amino-acid contact information in the predicted models. The three most relevant C-terminal domain structures then were attached by docking to the crystallographic NM-domain and the models are displayed in Fig. 4.

**Figure 5:**
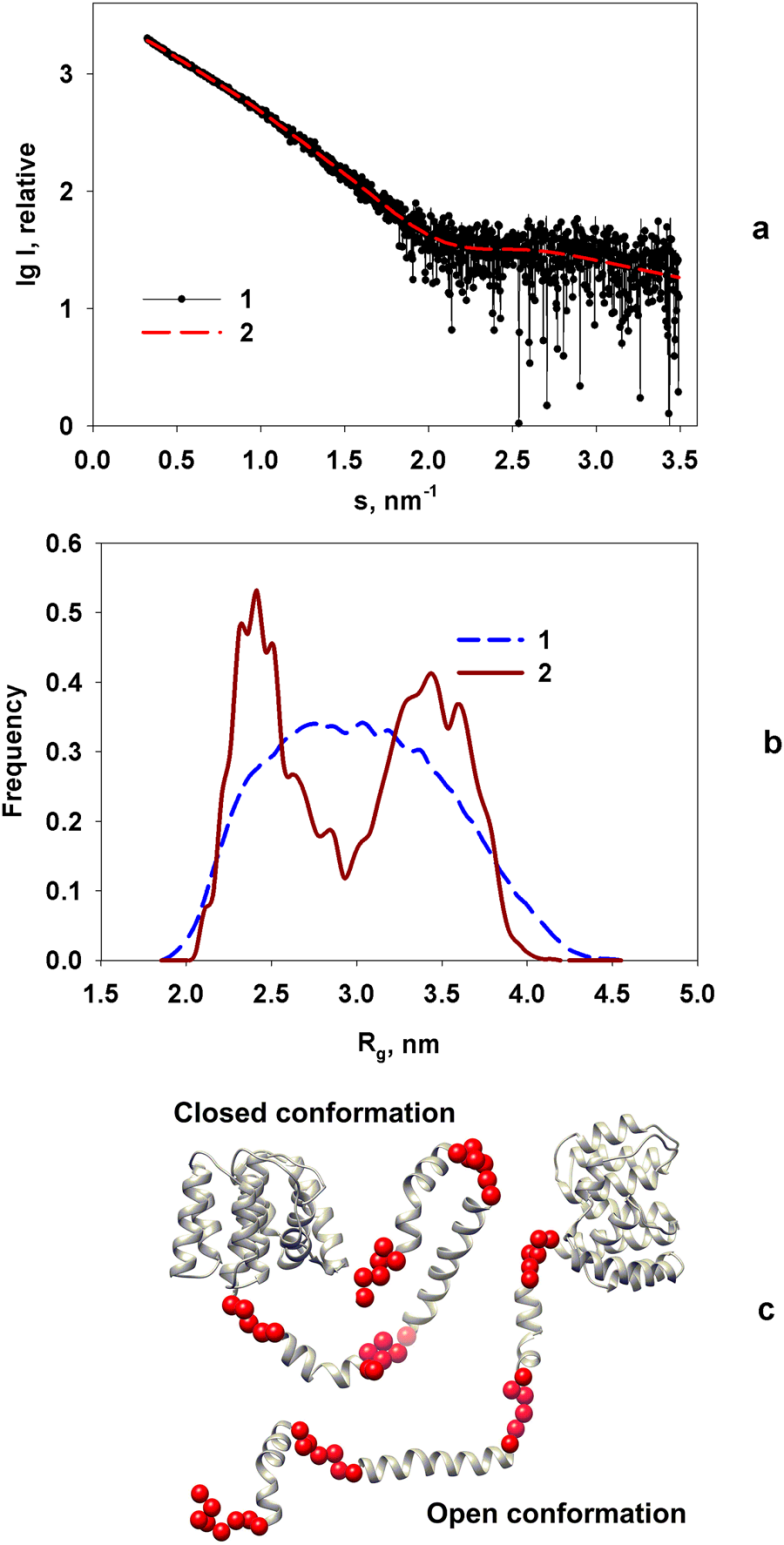
Analysis of flexibility of the full-length M1 by EOM. (**a)** Experimental SAXS data at pH 4.0 (1) and the scattering from the selected ensemble (2). **b)** *R*_*g*_ distributions, (random pool (1), selected ensemble (2)). (**c)** Typical open and closed conformation corresponding to the peaks of *R*_*g*_ distribution of the selected ensemble.

All selected models C1, C2 and C3 do not fit the experimental data well (Fig. 4, χ^2^ = 1.7–2.0) suggesting differences between the overall solution conformation and predicted rigid bodies where the flexibility and mobility of the C-terminal domain are not considered. To account for the flexibility, an ensemble optimization method (EOM)^47^ was applied. Here, possible conformations of the full-length M1 were allowed keeping the rigidity of the predicted C-terminal domain α-helices, but allowing flexibility and mobility of the linkers connecting them (Fig. 5).

The ensembles of M1 selected from initial pool of random structures provide a good fit to the experimental data with *χ* ^*2*^ ≈ 0.8 (Fig. 5a). The *R*_*g*_ distribution of the selected ensemble revealed two peaks, which correspond to the most probable conformations of the full-length M1 protein (Fig. 5b). Due to their shapes we called these conformations “open” and “closed” (Fig. 5c), respectively, reflecting a degree of C-terminal domain folding. These conformations are typical for the M1 protein at all pH values. The average *R*_*g*_ value over the ensemble (2.9–3.0 nm) coincides well with that obtained by Guinier analysis and also with that reported in^14^. The extent of the C-terminal domain spans the range between 2 nm and 9 nm with the most populated length of about 6–7 nm.

### Shape analysis of the M1 self-assemblies

**Figure 6:**
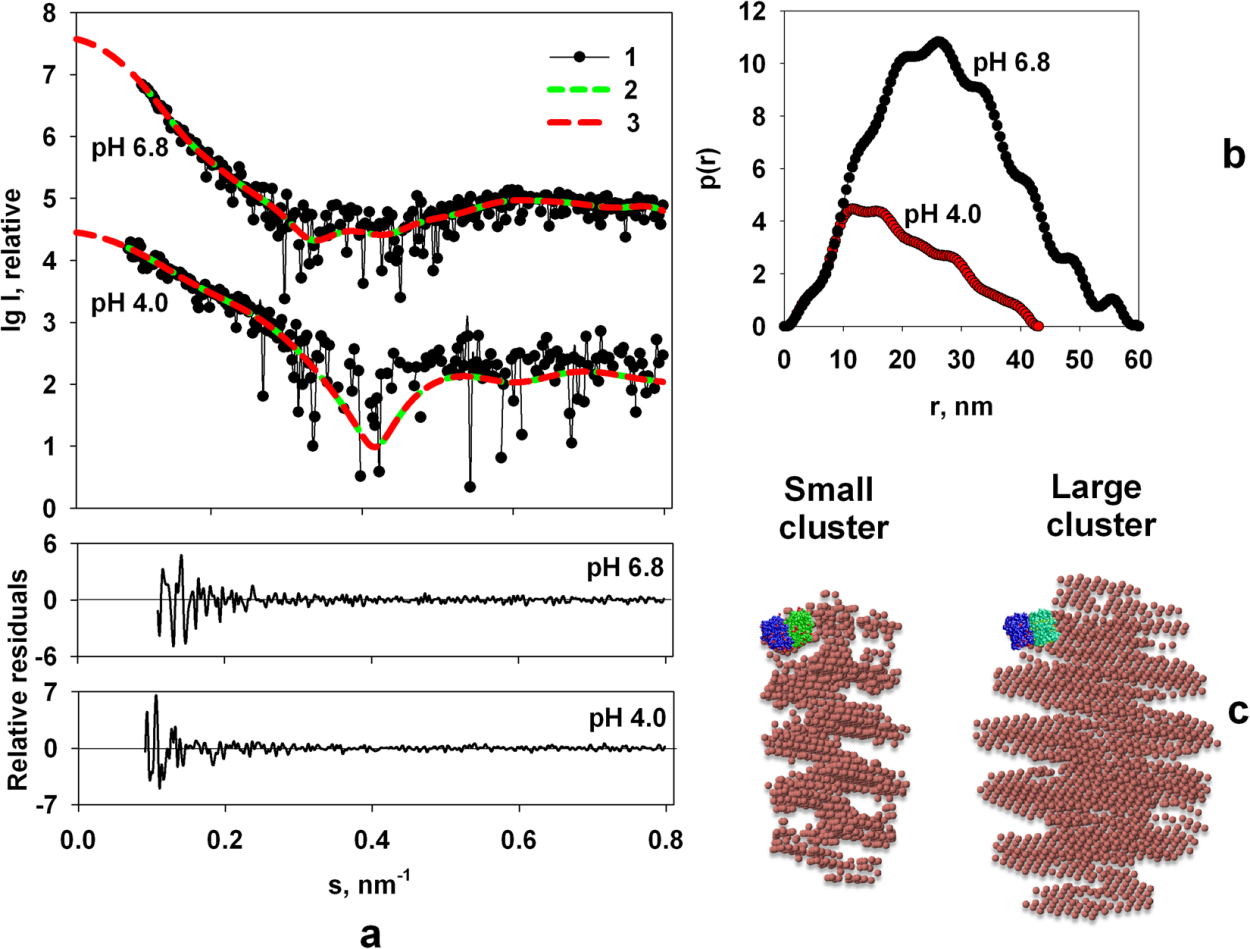
*Ab initio* shape restoration of the M1 self-assemblies at different pH values. (**a)** Difference curves corresponding to the scattering from the clusters (1), the scattering from the *ab initio* DAMMIN models (2), the transformed from *p*(*r*) and extrapolated to zero scattering angle intensities (3) at pH 4.0 and 6.8, respectively; the two lower panels are the residual differences between the experimental data and the scattering from the models calculated for pH 4.0 and 6.8, respectively. (**b)** Distance distribution functions of the clusters. (**c)** Typical reconstructed models of the clusters by DAMMIN. The crystallographic dimers of the NM-domain of the M1 protein at pH 4.0 and 6.8 aligned using MASSHA are shown in blue/green color for size comparison: PDB entry 1AA7 for acidic pH (small cluster) and PDB entry 1EA3 for neutral pH (large cluster).

To obtain the scattering from the clusters, the computed scattering from the *ab initio* model of individual M1 particles was subtracted from the experimental data of the full-length protein. The difference curves at different pH values were used for the structural analysis of the clusters (Fig. 6). Both of the *p*(*r*) distributions of the M1 clusters display repeating shoulders reflecting a regular internal structure. The *ab initio* shape of the clusters was reconstructed using DAMMIN, whereby multiple runs yielded reproducible results indicating a stable shape restoration. Typical *ab initio* shapes presented in Fig. 6c show that the M1 clusters have similar quasi-helical organization at different pH. Interestingly, the initial parts of the *p*(*r*) functions for *r* < 10 nm of the two clusters coincide well with each other. It means that the basic building blocks of the clusters are the same, and, most likely, they are М1 protein molecules densely packed in the same way as it was observed in the helical scaffold of M1 in the influenza virions^21,24^. To fit the NM-domain of the M1 monomer to the cluster structures and to compare their sizes on the same scale we utilized program MASSHA^65^.

Given the *ab initio* models of the clusters and of the individual M1 monomers, the experimental SAXS data from M1 containing these species were fitted by linear combinations of their scattering patterns to get volume fractions of the components using OLIGOMER^49^.

As one can see from the Table 1, pH 5.9 is some kind of a critical point when the process of the M1 multimerization in solution becomes favoured. The same pH value is the threshold for the dissociation of M1 from viral RNP^6^, leading to the loss of rigidity of virion^9^ and priming of the viral core for further RNA release. The helix-like shapes could be treated as pre-matrix protein superstructures or virus-like particles, whose formation is an intrinsic biological property of M1. Most importantly, this process is reversible, and at pH about 6.0 binding between M1 molecules starts to be more pronounced, most probably due to the change in interaction between C-terminal domains^10^.

**Table 1:** Volume fraction of the individual M1 macromolecules and clusters in solution.

pH	Mono, %	Small clusters, %	Large clusters, %
6.8	44	0.0	56.
6.4	72	0.0	28
5.9	81	4.0	15
5.2	94	6.0	0.0
4.7	99	1.0	0.0
4.0	98	2.0	0.0

### Change in M1-lipid interactions

**Figure 7:**
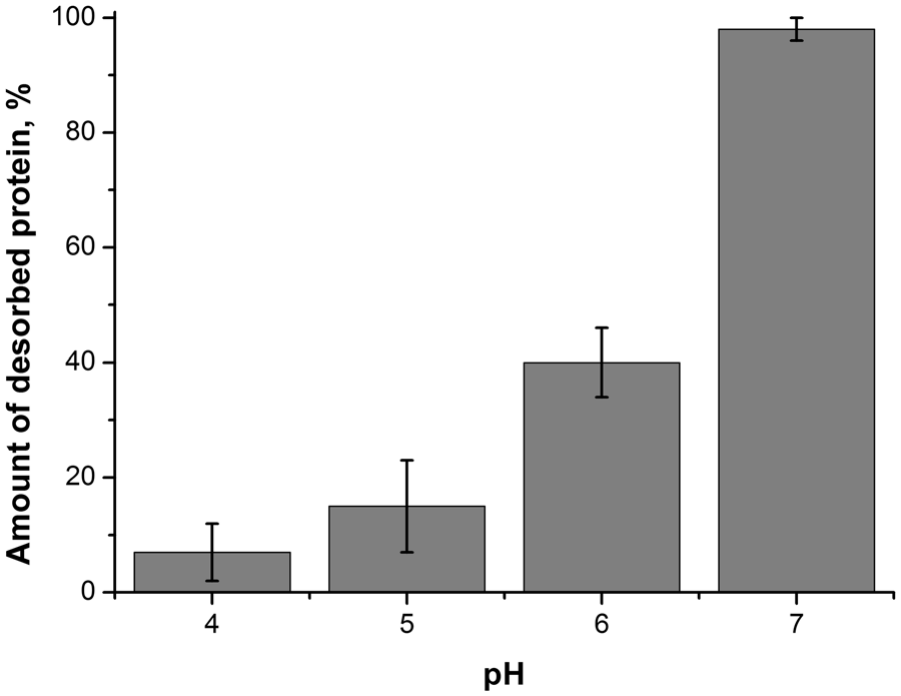
Amount of M1 protein desorbed from a negatively charged BLM after perfusion with protein-free working buffer solution at different pH. Bars represent the SD for three independent measurements. Initially, protein was adsorbed in concentration of 2 × 10^−3^ mg/ml. BLM composition is 30% DPhPS and 70% of DPhPC.

Analysis of kinetics of M1 adsorption on negatively charged BLM by IFC technique allowed us to assess the change of M1 binding to the membrane. In^10^ we showed that M1 protein adsorption is reversible at pH 7.1, however, this does not hold at lower pH. Figure 7 presents the percent of M1 protein molecules desorbed from the BLM at different pH. One can see that at pH 4 and pH 5 adsorption of M1 is almost fully irreversible, while at pH 6 nearly a half of the adsorbed protein was removed by perfusion with protein-free working buffer solution. This result correlates well with the predicted change of M1 self-association with pH (Table 1). Therefore, not only the protein-protein interactions in M1 scaffold but also M1 binding to the lipid membrane starts to change at a pH of around 6.0.

### AFM of M1 adsorption at different conditions

**Figure 8:**
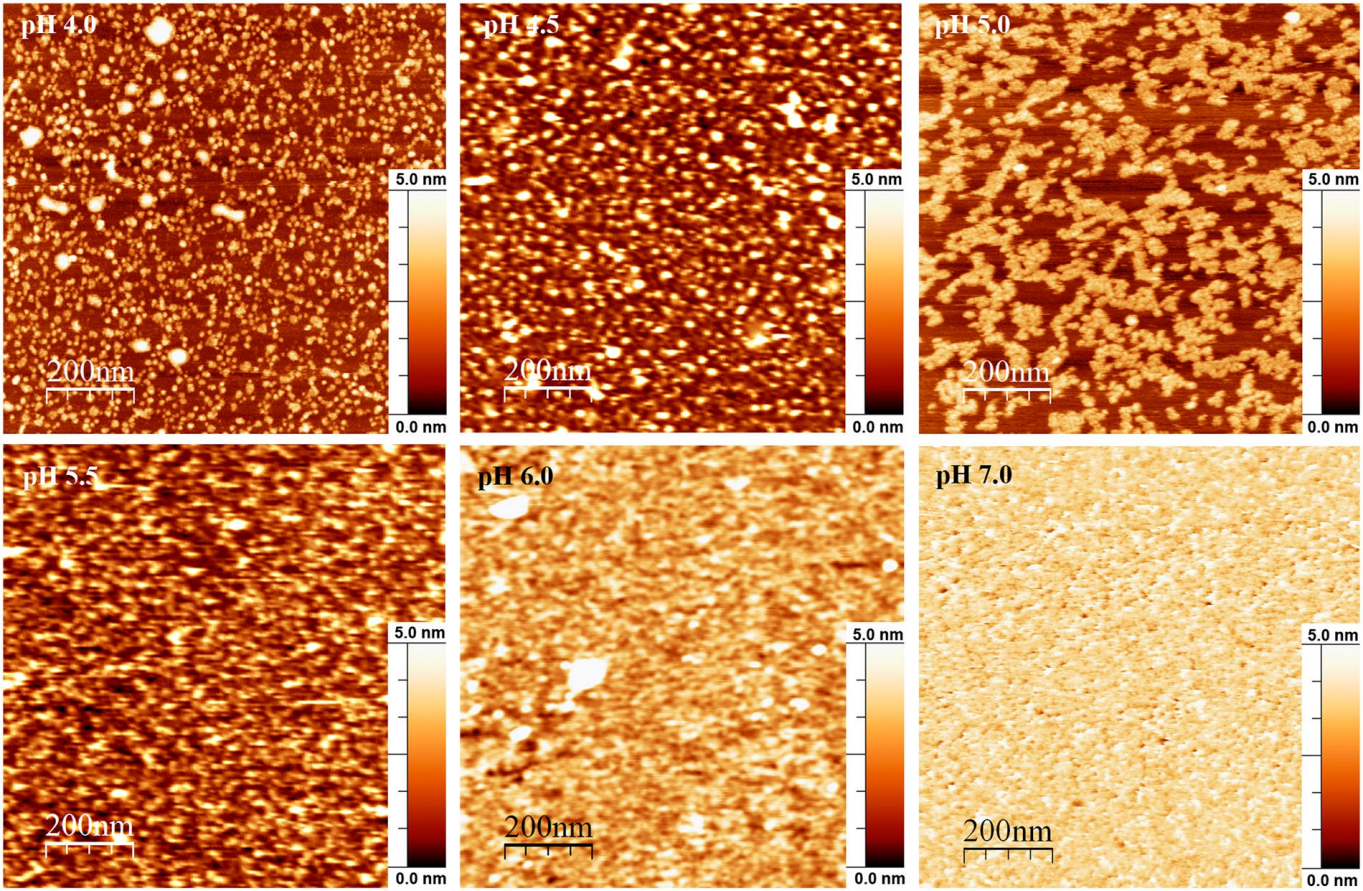
AFM topography images of M1 adsorbed at the mica surface at different pH values of the working buffer solution. Protein concentration was 2 × 10^−3^ mg/ml. Bright spots on the images are possible protein aggregates. Full z-scale is 5 nm.

In^10^ we demonstrated that the formation and destruction of the dense M1 monolayer on the lipid membrane is predominantly determined by electrostatic interactions. Moreover, this process runs similarly on the bare mica which surface charge density (−0.032 C/m^2^ for physiological range of ionic strengths)^66^ is very close to that of the inner leaflet of the viral lipid membrane^67,68^. Since solid mica surface provides more detailed topography images of the adsorbed protein layer, we decided to use it for study of the change of the protein layer density with pH (see Fig. 8).

**Figure 9:**
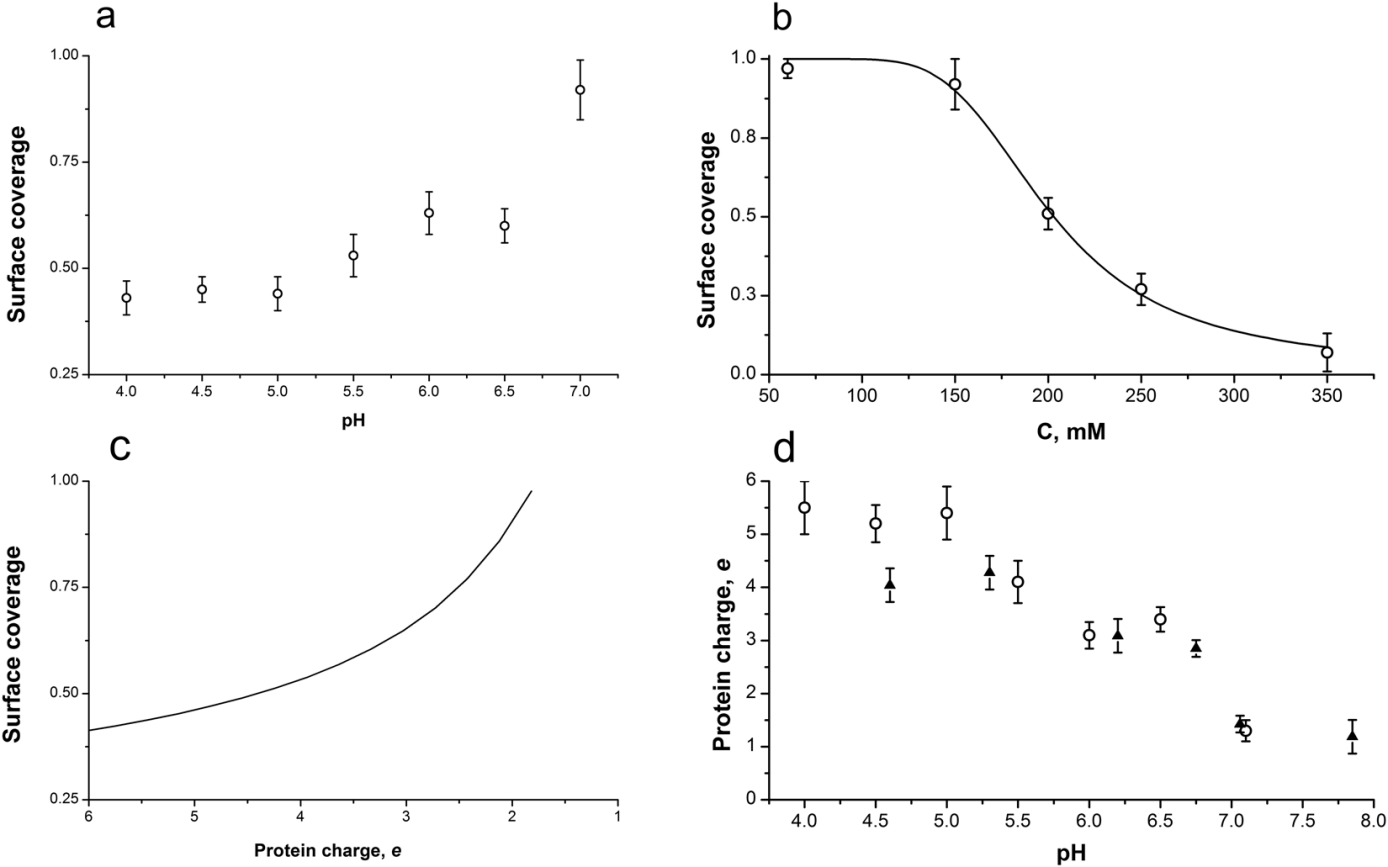
Calculation of the dependence of M1 charge on pH. (**a)** Dependence of the surface coverage of mica with M1 protein on pH of the working buffer solution. Protein concentration was 2 × 10^−3^ mg/ml. All data points are averaged from five independent AFM images. (**b)** Dependence of the surface coverage of mica with M1 protein on electrolyte concentration. Protein concentration was 2 × 10^−3^ mg/ml, pH of working buffer solution was equal to 7.1. All data points are averaged from five independent AFM images. Solid line is the fit of the experimental data with the Eq. (11) with the parameters *r*_0_ = 1.9 nm, *ε* = 10, *E*_*h*_ = 5.2 ± 0.7 *k*_*B*_ *T*, *q* = 1.3 ± 0.2 elementary charges. (**c)** Calculated from the Eq. 11 dependence of the surface coverage of mica with M1 protein on protein charge. (**d)** Dependence of the protein charge on the pH of the working buffer solution. *circles* – values of the protein charge obtained by combination of plots (**a**) and (**c**); *triangles* – the data obtained from electrophoresis experiments in 5 mM NaCl, 2.5 mM MES buffer with M1 concentration of 0.05 mg/ml by Eq. 12 for *r*_0_ = 2.9 nm, *ε* = 80.

We selected the bulk concentration of M1 to be sufficient for the dense monolayer at pH 7.0^10^, and used it at all pH. The surface coverage as a function of pH is presented in Fig. 9a; as expected from the electrostatic nature of M1 adsorption, the density of the protein layer decreased with ionic strength (Fig. 9b).

To extract the protein characteristics and parameters of protein-protein and protein-surface interactions, we built a statistical model of the observed processes. We considered individual protein molecules as spherical particles with the charge *q* and the radius *r*_0_. The free energy, *F*, of the interaction of charged particles with the surface can be written as:

5 $$F=x\cdot (\frac{{E}_{h}+{E}_{el}}{{k}_{B}T})+x\,{\rm{l}}{\rm{n}}(x)+(1-x)\,{\rm{l}}{\rm{n}}(1-x),$$

where *x* is the fraction of the surface covered by protein, *E*_*h*_ is the charge-independent energy of protein hydration repulsion from the surface and *E*_*el*_ is the electrostatic attraction, *k*_*B*_ is the Boltzmann’s constant and *T* is the absolute temperature. In case of charged protein molecules of finite size Eq. 5 should be modified by introducing effective surface coverage $\tilde{x}=x/\theta$, where *θ* reflects the ratio of protein cross-section, *r*_0_, to the effective cross-section, *r*_1_, caused by electrostatic repulsion between charged protein molecules, $\theta ={r}_{0}^{2}/{r}_{1}^{2}$:

6 $$F=\theta \cdot [\tilde{x}\cdot (\frac{{E}_{h}+{E}_{el}}{{k}_{B}T})+\tilde{x}\,\mathrm{ln}(\tilde{x})+(1-\tilde{x})\mathrm{ln}(1-\tilde{x})].$$

Minimizing this energy by $\tilde{x}$ we obtain the equilibrium surface coverage:

7 $$x=\theta \cdot {(1+{e}^{\frac{{E}_{h}+{E}_{el}}{{k}_{B}T}})}^{-1}.$$

According to Derjaduin-Landau-Verwey-Overbeek (DLVO) theory^69,70^, electrostatic part of the energy of interaction of the charged spherical particle with the radius *r*_0_ and the charge *q* with the oppositely charged surface could be written in the following form:

8 $${E}_{el}=\frac{q{\sigma }_{0}{\lambda }_{D}^{2}}{\varepsilon {\varepsilon }_{0}{r}_{0}}{e}^{-h/{\lambda }_{D}},$$

where *σ*_0_ is the surface charge density of the surface; *λ*_*D*_ is the Debye length, given by eq.

$${\lambda }_{D}=\sqrt{\frac{\varepsilon {\varepsilon }_{0}{k}_{B}T}{{e}^{2}\sum {c}_{i}{z}_{i}^{2}}};$$

*h* is the distance between particle and the surface; *ε* and *ε*_0_ are dielectric permittivity and electric constant, respectively; *e* is the elementary charge, *c*_*i*_ and *z*_*i*_ are concentration and valence of electrolyte’s ions, respectively. Since protein molecules adsorb to the mica surface we can put *h* = 0 and for distances of protein radius from the mica surface we should take *ε* = 10^71^.

To estimate the effective protein radius *r*_1_, which takes into account electrostatic repulsion between individual proteins, we can rewrite Eq. (8) for the case of two spherical particles in a close contact:

9 $${E}_{1}=\frac{{q}^{2}{\lambda }_{D}^{2}}{8\pi \varepsilon {\varepsilon }_{0}{r}_{0}^{3}}{e}^{-2({r}_{1}-{r}_{0})/{\lambda }_{D}},$$

The radius of the protein molecule could be obtained from the assumption that the energy of electrostatic repulsion should be not less than the energy of thermal motion, *k*_*B*_ *T*, as for Bjerrum length determination, so we can find *r*_1_ as:

10 $${r}_{1}={r}_{0}-\frac{{\lambda }_{D}}{2}\,\mathrm{ln}(\frac{8\pi \varepsilon {\varepsilon }_{0}{r}_{0}^{3}{k}_{B}T}{{\lambda }_{D}^{2}{q}^{2}}).$$

It is noteworthy that the value of *r*_1_ should always be greater than *r*_0._ Otherwise we should take *r*_1_ = *r*_0_. Combining Eq. 8–10, we obtained the equilibrium surface coverage:

11 $$x=\frac{1}{{(\frac{{r}_{1}}{{r}_{0}})}^{2}(1+\exp [\frac{1}{{k}_{B}T}({E}_{h}-\frac{q{\sigma }_{0}{\lambda }_{D}^{2}}{\varepsilon {\varepsilon }_{0}{r}_{0}})])}$$

Our SAXS data demonstrated that the structure of M1 monomer is largely preserved with pH, and the radius of gyration of the compact part of M1 protein (N-terminal domain) is 1.9 nm. As we show in^10^, the flexible C-terminal domain should be aligned perpendicular to the surface since it is responsible for M1-RNP binding^6^. Therefore, for the in-layer interactions between M1 molecules on the surface we took *r*_0_ = 1.9 nm. As a result, in Eq. 11 we have only two parameters: protein charge *q* and the charge-independent energy of protein hydration repulsion from the surface, *E*_*h*_. Performing the fit of the data presented in Fig. 9b we obtained the value of *E*_*h*_ = 5.2 ± 0.7 *k*_*B*_ *T* and the protein charge at pH 7.1 equal to *q* = 1.3 ± 0.2 elementary charges. This protein charge corresponds to the surface charge density of 0.5 ± 0.1 μC/cm^2^, which is the same as the value obtained in experiments with protein adsorption on negatively charged lipid membranes^72^. Substituting the value of *E*_*h*_ into Eq. 11 we calculated the dependence of surface coverage on protein charge (Fig. 9c). Combination of Fig. 9a,c allows one to obtain the pH dependence of the protein charge (Fig. 9d, circles). From the dependence of the protein charge on the pH (Fig. 9d) one can see two characteristic regions in this plot, at pH 5.0–5.5 and at pH 6.5–7.0 that may correspond to distinct changes in the surface potential of the protein caused by the en masse protonation and de-protonation of charged amino acid side chains. For example, the first region is close to the value of pH 5.0 that has been previously reported as the possible isoelectric point of the M1 protein^73^ while the second region at near-neutral pH may be connected with the dissociation of M1 from RNP which takes place at pH around 6.0^6^.

To independently verify these results and our model assumption we conducted zeta-potential measurements in solutions of M1 with different pH values. Assuming spherical particles, the protein charge *q* can be calculated from the zeta-potential *ϕ* by

12 $$q=(1+{r}_{0}/{\lambda }_{D})4\pi \varepsilon {\varepsilon }_{0}{r}_{0}\varphi .$$

Here we also took the value of protein size, *r*_0_, equal to the radius of gyration of N-terminal domain, *r*_0_ = 1.9 nm. The obtained dependence of the protein charge (Fig. 9d, triangles) shows a good agreement with the results calculated from our electrostatic model, demonstrating the validity of the our assumptions.

### Model of M1 self-assembly in the solution

We have shown that M1 protein has a tendency to self-associate in helical structures at pH 4.7^14^. Similar structures of the M1 scaffold is reported to be part of the intact viral particle^20,21^, so they should exist also at pH 7. From our SAXS data on M1 the pH increase leads to M1 molecules self-assemble into helical structures (Table 1). To obtain the expression for the volume fraction of multimerized M1 molecules, we considered the formation of protein helix as a linear polymerization process. The elementary step of the reaction is:

13 $$ \sim {R}_{n}+M\underset{\mathop{\longleftarrow }\limits_{{k}_{d}}}{\overset{{k}_{p}}{\longrightarrow }} \sim {R}_{n+1},$$

where *k*_*p*_ and *k*_*d*_ are polymerization (multimerization) and disintegration rate constants, respectively. In an equilibrium case volume fraction of proteins in monomeric form should be $x=\frac{{k}_{d}}{{k}_{p}}$. Using Arrhenius form for rate constants and transition state theory, we obtained:

14 $$x=\frac{{k}_{d}}{{k}_{p}}=\frac{\exp (\frac{{\rm{\Delta }}{S}_{d}^{\ne }}{{k}_{B}})}{\exp (\frac{{\rm{\Delta }}{S}_{p}^{\ne }}{{k}_{B}})}\exp (\frac{{E}_{p}-{E}_{m}}{{k}_{B}T})=\exp (\frac{{\rm{\Delta }}{S}^{0}}{{k}_{B}})\exp (\frac{{E}_{p}-{E}_{m}}{{k}_{B}T})=\frac{1}{{x}_{0}}\exp (\frac{{E}_{p}-{E}_{m}}{{k}_{B}T}),$$

where ${\rm{\Delta }}{S}_{p}^{\ne }$ and ${\rm{\Delta }}{S}_{d}^{\ne }$ are changes of entropy between monomer-transition state and polymer-transition state, respectively, ${\rm{\Delta }}{S}^{0}$ is the entropy of the monomer standard state, *E*_*p*_ and *E*_*m*_ are free energies of polymer and monomer, correspondingly, *x*_0_ is the initial volume fraction of monomers in solution. It is noteworthy that this approach is eligible only for the case of large protein clusters, so we can use Eq. (14) only in the range of pH from 5.9 to 6.8 (see Table 1), taking the volume fraction of monomers equal to 1 for other values of pH.

Similar to the case of protein adsorption to the mica surface we can take the difference between energies of M1 monomers and helices in solution as a sum of charge-dependent and charge-independent energies, thus:

15 $${E}_{p}-{E}_{m}=-{E}_{0}+\frac{{\lambda }_{D}^{2}{q}^{2}}{8\pi \varepsilon {\varepsilon }_{0}{r}_{0}^{3}},$$

where *E*_0_ is the charge-independent part of the protein-protein interaction.

**Figure 10:**
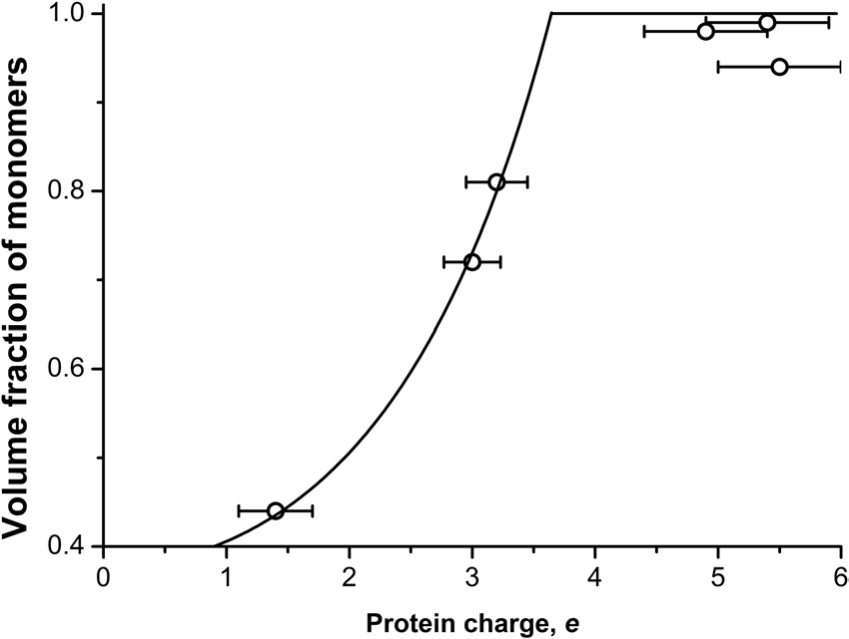
Volume fraction of M1 monomers in solution depending on the protein charge. Solid line is the fit of experimental data with Eq. (14). Protein concentration was 1.47 mg/ml in 100 mM NaCl, 50 mM MES buffer.

Combining Eq. (14) with Eq. (15) and using the obtained dependence of M1 protein charge on pH (Fig. 9d) we fitted the experimental data of protein multimerization in solution (Fig. 10) and obtained the values *E*_0_ = 7.5 ± 0.1 *k*_*B*_ *T* and *r*_0_ = 1.8 ± 0.1 nm. The latter value, which agrees with the radius of gyration of NM-domain, once again proves our hypothesis that the compact N-terminal domain of M1 in responsible in electrostatic (and, consequently, pH-dependent) protein-protein interactions. This result is consistent with the observations of^23^ where authors show that individual C-terminal parts of M1 are dimers both in acidic and neutral conditions, while the interactions between N-terminal domains change dramatically. The obtained charge independent energy *E*_0_ = 7.5 ± 0.1 *k*_*B*_ *T* is comparable with the energy of single hydrogen bond^74^, so it may be the case that M1 molecules stabilize into helical multimers forming such bonds between C-terminal domains. According these results, protein multimerization begins when the charge of the molecule is equal to approximately 3.5 elementary charges that corresponds to pH 5.9. It is reported in^6^ that at pH 6.0 M1 protein dissociates from viral RNP, which it binds by C-terminal domain. The results presented in^19^ as well as our previous observations also suggest that this domain is responsible for protein oligomerization. Therefore, the connection with RNP and the binding between M1 molecules start to break at pH 6, consistent with what was shown in^10^ for the dissociation between the first and second adsorbed protein layer.

**Figure 11:**
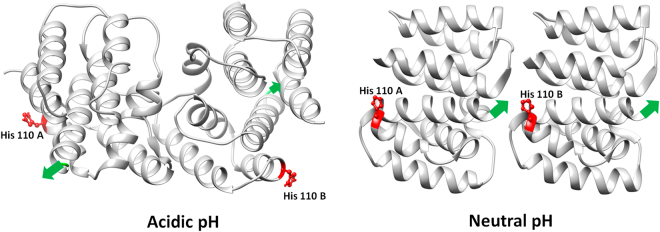
Mutual orientation of the NM-domains in crystal structures at different pH: PDB entry 1AA7 for acidic pH and PDB entry 1EA3 for neutral pH. The positions of the histidines in the M1 protein crystallographic dimers are marked by red. Green arrows mark localization and direction of the C-terminal domains.

The mutual orientation of the NM-domains in the crystal structure at different pH can serve as a prototype of the orientation of the protein in the helical matrix layer (see Fig. 11).

**Figure 12:**
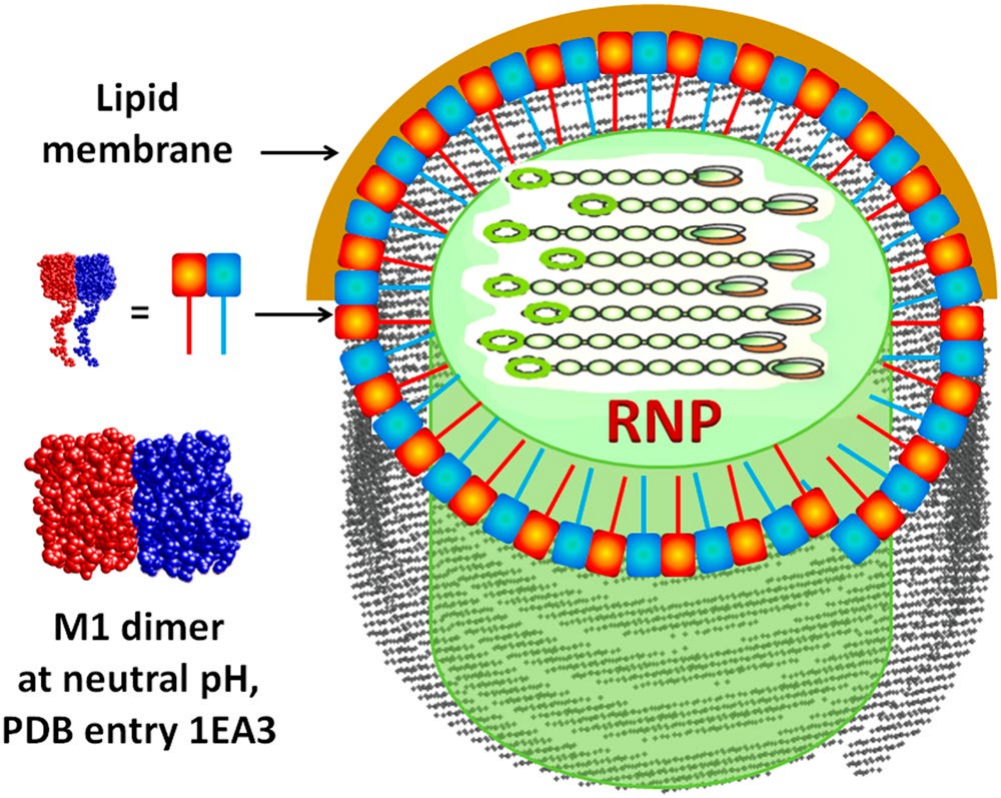
Schematic representation of the most probable orientation of M1 molecules within the virion at pH 7.0.

First, one can see that at acidic pH, the flexible C-terminal domains are located on the opposite sides of the protein molecules within the dimer and protrude in different directions, while at neutral pH, the C-terminal domains are on the same side protruding in the same direction. In neutral medium the binding of M1 to the lipid membrane possibly occurs via the globular part of the protein, while C-terminal domain is responsible for the interaction with viral RNP^6^. We can hypothesize that the same structure is preserved in the M1 clusters observed in our SAXS measurements such that in neutral medium all C-terminal domains should point to the axis of the helical cluster (Fig. 12).

When the pH decreases a transition takes place to the M1 configuration with C-terminal domains protruding in different directions, corresponding dissociation of the M1 clusters, as occurs when the viral protein scaffold is exposed to an acidic medium in the late endosome. As we observed the most pronounced change of the amount of M1 clusters in the solution at pH around 6, histidine, the only amino acid with pKa of the side chain of around 6, should play a major role in such transition. If so, even at pH 4.0 approximately one percent of histidines should be in dissociated form that coincides with the amount of small clusters in acidic medium (see Table 1).

**Figure 13:**
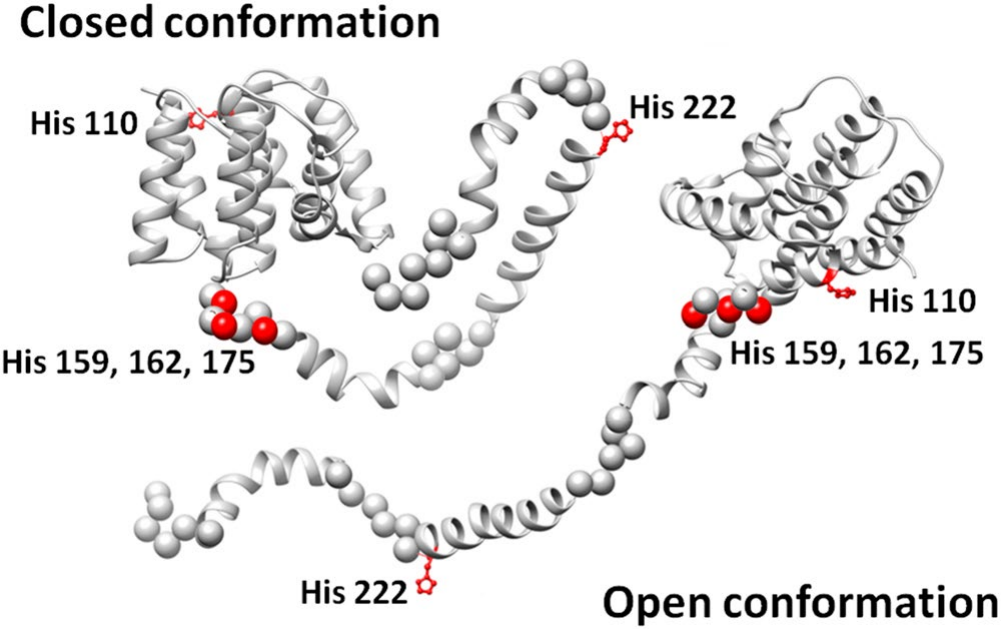
Location of histidine residues in the M1 monomer. Red spheres indicate the location of histidines in regions recovered by hybrid methods (EOM) from small-angle scattering data. Location of His 222 was predicted by ROSETTA modeling software^50^ due to the position of this amino acid on α-helix.

Analysis of M1 structure revealed five histidines, one in NM-domain and the others in the C-terminal part (see Fig. 13). The histidines located in the NM-domain are not proximal at both pH 7.0 and pH 4.0 (see Fig. 11), as follows from the crystallographic structures (PDB entry 1AA7 for acidic pH and PDB entry 1EA3 for neutral pH). Thus, most probably, they contribute to the dissociation of M1 from the lipid layer.

Other histidines in the M1 molecule are located in the partly disordered C-terminal domain (Fig. 13). We cannot identify the amino acids responsible for M1-RNP and M1-M1 binding but one can hypothesize that His 222 most probably interacts with RNP since it is located almost at the C-terminus of the molecule and away from other histidines. Histidines 159, 162 and 175 stand close to each other and to the globular NM-domain. Therefore, we assume that they orchestrate M1-M1 interactions within helical clusters through hydrogen bonds with opposing amino acids. This would be in line with the predictions of our theoretical model showing that the charge-dependent M1-M1 interactions should occur via NM-domains while charge-independent binding utilizes hydrogen bonds. Thus, flexible C-terminal domain of M1 governs both M1-RNP interactions and the formation of the viral helical scaffold.

## Conclusion

In the present study, we characterized the structure of the influenza virus matrix protein M1 in the pH range of 4.0 to 6.8. The minimal structural unit for the protein is a monomer consisting of the compact N-terminal domain and partially flexible C-terminal tail. The overall size of the monomer as well as the conformation of its globular N-terminal part (NM-domain) is preserved in the entire studied pH range. At the same time, the oligomerization state of the protein and its binding to the lipid bilayer change dramatically with pH. At low pH conditions, M1 protein exists in solution predominantly as a monomer, with very low fraction of small helical oligomers. The binding of such monomers to the lipid membrane is rather strong, as we demonstrated earlier^10^. In a neutral milieu, M1 molecules form large oligomers with the layered structure close to the protein scaffold of influenza virions. Simultaneously, their interaction with lipids becomes weaker. The threshold pH value for the change of both M1 oligomerization state and its membrane binding lies near pH 6.0, which is reported to be the point of loss of binding of M1 with the viral RNP at the priming stage^6^ and corresponding softening of the viral particle itself^9^. We hypothesized that both M1-M1 and M1-lipid interactions are governed by electrostatic forces and developed a model for the self-assembly of M1 protein at different values of pH. The model yields a good agreement with the results of direct measurements of zeta-potential and protein oligomerization state in solution. The charge-independent part of the binding energy of M1 molecules suggests the formation of hydrogen bonds between molecules, which break by Coulomb repulsion below pH 6.0. This threshold value of pH points to the possible role of histidines predominantly located in the C-terminal domain in the self-organization of the M1 helical scaffold. The size of the charged part of the molecule obtained from the model corresponds to the radius of the NM-domain pointing to the fact that this domain provides the main contribution to the pH dependence of M1 oligomerization. In^23^ the authors show that C-terminal domain alone should be in a form of dimer even in an acidic environment, while N-terminal domain changes its oligomerization state with pH. Our data^14^ suggest that NM-domain is a monomer in solution at low pH. Combining these results with the data of the present study, we can conclude that the C-terminal domain is responsible for the formation of hydrogen bonds between M1 monomers, while the N-terminal part regulates the protein multimerization and its membrane binding at different pH.
